# Subconjunctival dirofilariasis in a healthy subject: a case report

**DOI:** 10.1186/s12348-024-00447-5

**Published:** 2024-12-18

**Authors:** Amirhossein Aghajani, Mohammad Taher Rajabi, Seyed Mohsen Rafizadeh, Elham Rahmanikhah, Reza Samiee, Mohammad Reza Abdol Homayuni, Amin Zand

**Affiliations:** 1 Department of Oculo-Facial Plastic and Reconstructive Surgery, Farabi Eye Hospital, Tehran University of Medical Sciences, Tehran, Iran; 2 School of Medicine, Tehran University of Medical Sciences, Tehran, Iran

**Keywords:** *Dirofilaria*, Ocular Dirofilariasis, Ocular parasite, Ocular Adnexa

## Abstract

**Purpose:**

To report a case of subconjunctival dirofilariasis presenting as an inflammatory mass-like lesion in a healthy individual.

**Case presentation:**

A 20-year-old male with a recent history of wildlife exposure in a rural village presented with an inflammatory subconjunctival mass in his left eye. The lesion was surgically excised, and histopathological examination confirmed the presence of an immature nematode of *Dirofilaria immitis*. The patient was treated with oral ivermectin for two weeks postoperatively. After two months, the lesion had almost completely resolved, and no recurrence was observed during the 6-month follow-up.

**Conclusions:**

Given the rarity of ocular dirofilariasis and its diverse presentations in different ocular and adnexal structures, prompt and complete excision followed by meticulous histopathological evaluation is crucial to guide appropriate management and achieve favorable outcomes.

## Introduction

Filariasis is a parasitic disease that affects both domestic and wild animals worldwide, with canines serving as the primary reservoir hosts. While primarily an animal disease, filariasis can occasionally be transmitted to humans as a zoonotic infection through mosquito bites [1, 2]. The parasites are transmitted by mosquito vectors during the blood-borne microfilariae stage [3]. Clinically, filariasis can manifest as peripheral pulmonary or subcutaneous nodules and, in some cases, may even involve the heart [4, 5]. Rarely, the disease can affect periocular tissues and the eyes [1, 2].

Although human ocular dirofilariasis is uncommon, an increasing number of cases have been reported globally. Ocular involvement can present in various forms, including periorbital, subconjunctival, orbital, or intraocular infections [6]. Previous literature recommends the extraction of the parasite from the affected tissue as both a definitive diagnostic method and a treatment strategy [7].

The rarity of ocular manifestations of dirofilariasis, combined with variations in its presentation, can cause it to masquerade as other inflammatory ocular surface lesions, as demonstrated in our case [6]. In this report, we present the case of a healthy individual with a recent history of wildlife exposure who developed a subconjunctival inflammatory mass lesion. The lesion was subsequently diagnosed as ocular dirofilariasis following complete excision and pathological examination.

## Case report

This study adheres to the CARE guidelines [8]. All procedures were conducted in accordance with the principles outlined in the Declaration of Helsinki. Written informed consent for the publication of this report and related images was obtained from the patient, with all identifying details de-identified. Ethical approval for case reports was not required by the institutional review board.

A healthy 20-year-old male presented to the emergency department of Farabi Eye Hospital, Tehran, Iran, with a 2-week history of redness and a bulging mass on the ocular surface of his left eye. The patient had no significant ocular or medical history and reported no trauma or insect bites. He denied symptoms such as fever, chills, cough, vomiting, diarrhea, weight loss, rashes, or joint pain. The patient reported residing on a farm in a village near Jolfa Town in northwest Iran for three months, until one month before presentation. The area had fresh water sources and high humidity. During his stay, he had close contact with dogs that were not regularly examined by a veterinarian.

**Fig. 1:**
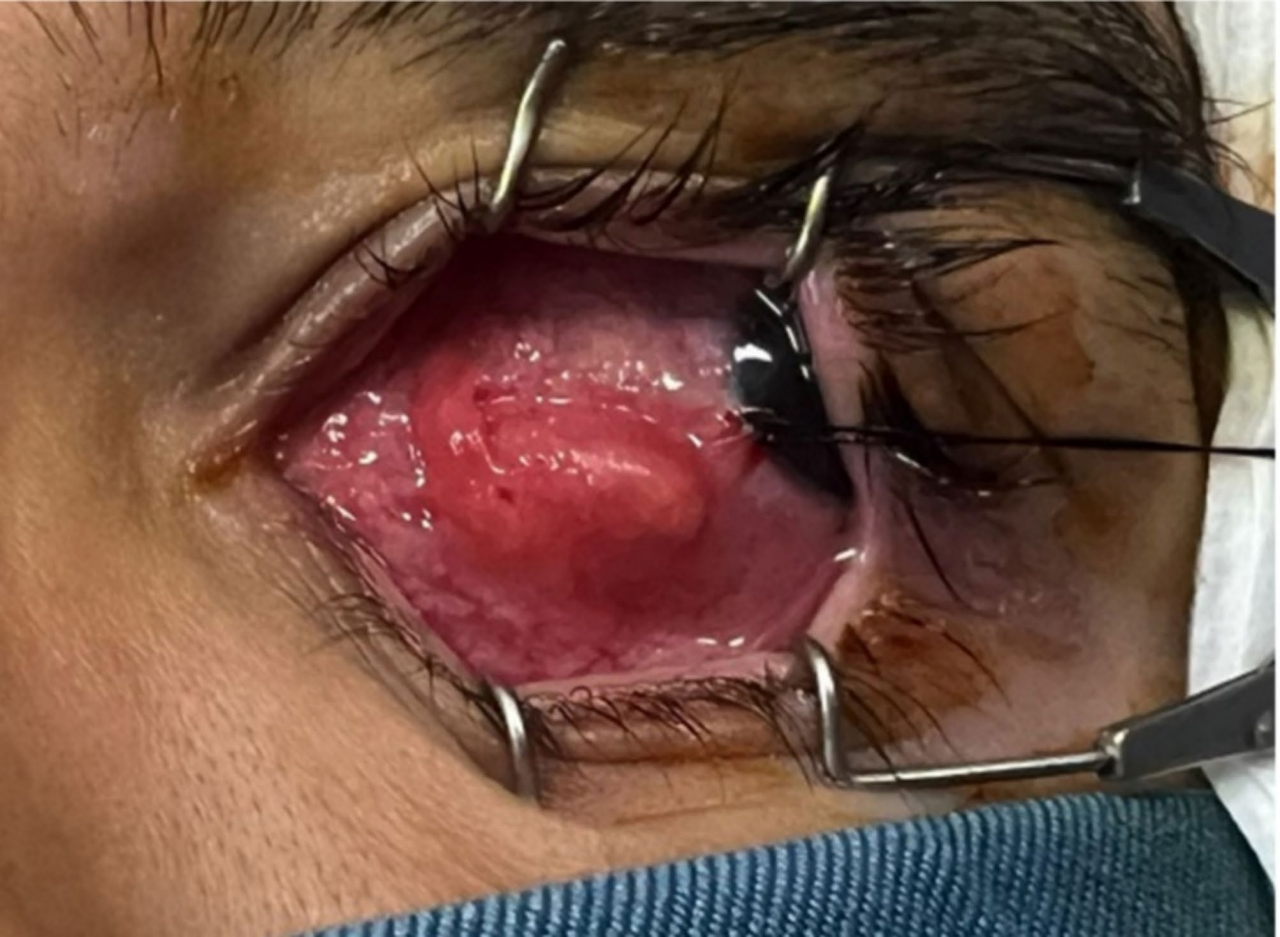
Inflammatory mass-like lesion located behind the nasal bulbar conjunctiva of the left eye, measuring approximately 6 × 10 mm in diameter (the globe is rotated temporally using a traction suture)

On examination, his best-corrected visual acuity was 20/20 in both eyes. Pupillary reactions were normal, with no relative afferent pupillary defect, and ocular movements were unrestricted. The eyelids appeared normal. Conjunctival injection was observed in the left eye. Slit-lamp examination revealed a bulging mass lesion within the nasal bulbar subconjunctival space, measuring 10 × 6 mm, without corneal involvement (Fig. 1). No anterior segment reaction was detected. A dilated fundus examination revealed clear vitreous and a normal retina and optic discs. Laboratory investigations, including a complete blood count with differential, erythrocyte sedimentation rate (ESR), and qualitative C-reactive protein, were unremarkable.

The patient was scheduled for surgical excision of the mass for further pathological investigation and appropriate management. The overlying conjunctiva and Tenon’s capsule were incised, and the lesion was dissected from the underlying sclera and completely excised.

**Fig. 2:**
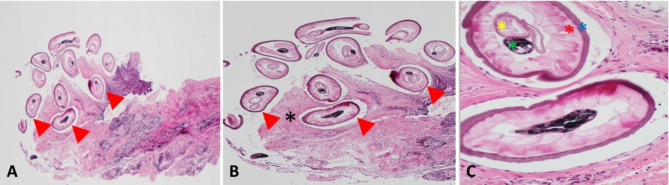
(**A, B**) Microscopic examination reveals a variably degenerated nematode with thick, multilayered cuticles (arrowheads) (Hematoxylin and Eosin [H&E] staining; ×40 [**A**] and ×100 [**B**] magnifications). Cuticles surrounded by eosinophilic necrosis (black asterisk).(**C**) Morphologic features of a female *Dirofilaria immitis*, including a multi-layered ridged cuticle (blue asterisk), tall and prominent muscle cells (red asterisk), coiled intestine (yellow asterisk), and coiled vagina (green asterisk) (H&E staining; ×400 magnification)

The specimen was sent to the pathology laboratory in formalin solution. Gross pathology revealed subconjunctival tissue with sections of a degenerated parasitic organism, along with chronic granulomatous inflammation, including coagulation necrosis surrounded by fibrous tissue and granulation. The parasite was identified as a nematode larva with thick, smooth cuticles (Fig. 2A-B). Further microscopic examination revealed prominent internal cuticular ridges, a thick cuticle, a well-developed muscular layer, an intestinal tube, and uterine tubules, consistent with an immature female nematode larva of *Dirofilaria immitis* (Fig. 2C).

**Fig. 3:**
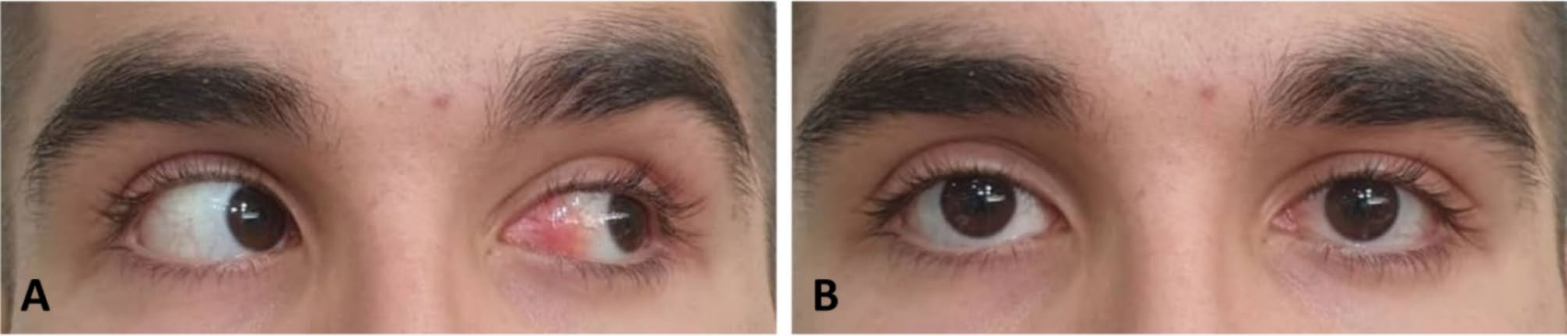
(**A, B**) Six months postoperatively, nearly complete healing is observed without recurrence of the lesion

Following the diagnosis of ocular dirofilariasis, the patient was referred to an infectious disease specialist who prescribed oral ivermectin, 15 mg daily, for two weeks. During subsequent follow-up visits every two weeks, the size of the lesion, inflammation, and redness gradually decreased. After two months, the lesion had nearly disappeared. No recurrence was observed at the 6-month follow-up (Fig. 3A-B).

## Discussion

*Dirofilaria* species are globally recognized zoonotic pathogens that can sporadically infect humans. The adult Dirofilaria nematode reproduces sexually in vertebrate hosts, primarily dogs and cats, and occasionally humans. Microfilariae (first-stage larvae) are ingested by intermediate hosts (e.g., mosquitoes or fleas) during blood feeding. Inside the vector, the larvae develop and mature into the infective third-stage larvae, which are transmitted to vertebrate hosts during subsequent blood meals [9]. The most common species infecting humans include *D. immitis* (a parasite of dogs), *D. tenuis* (a parasite of raccoons), and *D. repens* (a parasite of cats and dogs) [3]. Interactions between these pathogenic species and the endosymbiont bacterium *Wolbachia* may increase the likelihood of human infections [5, 10]. Risk factors for human infection include contact with wildlife, particularly cats and dogs, as well as environmental factors such as tropical climates, which enhance the feeding and breeding cycles of mosquito vectors, thereby increasing the pool of available filarial larvae [11]. However, in some cases, no clear risk factors can be identified from the patient’s history [6, 7]. In this case, the patient had prolonged exposure (approximately 3 months) to dogs on a village farm with the presence of fresh water and a high-humidity climate. These environmental conditions may have enhanced the feeding and breeding cycles of potential vector species (e.g., mosquitoes), thereby increasing the pool of available filarial larvae and the risk of disease transmission [12, 13].

*Dirofilariasis* can affect various parts of the human body, including the lungs, subcutaneous tissue, eyes and adnexa, heart, and testicles [3]. Ocular dirofilariasis is rare and can involve any structure of the eyes and adnexa [7]. The disease may mimic an abscess or soft tissue mass in ocular adnexa, necessitating definitive diagnosis by excision of the lesions and pathological evaluation [14]. In a review by Camacho et al. the most common sites of ocular dirofilariasis involvement were found to be the subconjunctiva, eyelid, orbit, anterior chamber, vitreous, and chorioretinal areas, respectively [6]. Similarly, in Iran, most ocular dirofilariasis cases have been reported with subconjunctival involvement, typically caused by *D. immitis* and *D. repens* [15–18]. In most cases, the disease does not lead to serious complications, but there are reports where untreated cases have progressed to rapid-onset orbital cellulitis or extensive chorioretinal damage, resulting in significant vision impairment [14]. Our patient, consistent with most reports, presented with subconjunctival involvement by *D. immitis*, the most commonly reported site of ocular involvement.

Previous studies have shown that only adult or degenerated immature juvenile nematodes infect humans, with microfilariae rarely reproducing in human hosts [3]. Camacho et al. reported a case involving an adult nematode behind the subconjunctival tissue [6]. Kalogeropoulos et al. and Mahesh et al. documented cases with immature nematodes in the eye and adnexa [7, 14]. In our case, we detected sections of a degenerated immature female *Dirofilaria* larva in the subconjunctival tissue. The infection triggered an inflammatory granulomatous reaction, characterized by infiltrating neutrophils, eosinophils, and foreign body giant cells. Chronic infection may further lead to granulomatous responses with calcification or abscess formation [6]. Similar pathological findings were observed in our case.

While eosinophilia and elevated inflammatory markers may suggest a parasitic infection and provide clues for residual disease after surgical excision, routine laboratory investigations are not typically recommended. Previous studies have indicated that systemic eosinophilia is observed in only up to 20% of humans infected by *Dirofilaria* [3, 6, 19].

A definitive diagnosis of dirofilariasis can only be made through surgical removal of the lesion followed by pathological assessment [7]. Identifying the parasite requires histological and morphological examination of the worm, with attention to its large muscle cells, wide lateral chords, and thick laminated cuticles. Species identification is achieved by examining the mature worm under a microscope. When only histopathological specimens are available, features such as the size and characteristics of the body wall—including cuticle thickness, structure, ridges, the number of lateral chords, and muscle cell type—can aid in differentiating the species in cases of deep tissue involvement [3, 6, 10, 20, 21]. PCR-based DNA analysis can also provide an accurate diagnosis when standard morphological assessment is not feasible due to inadequate worm conservation [20].

Prompt and complete surgical excision of the nematode is considered the treatment of choice and is usually curative [6, 7]. Due to the solitary nature of *Dirofilaria* and its lack of reproductive activity in humans, antihelminthic drugs are generally ineffective [22]. Therefore, systemic therapy with antinematodal drugs is not routinely required, though some reports have employed these drugs, including ivermectin, following surgical excision to reduce the chance of recurrence, particularly when residual disease or microfilaremia is suspected [3, 6]. Furthermore, oral ivermectin is recommended in cases of suspected systemic infection [18]. In our case, we treated the patient with ivermectin for two weeks following excision of the subconjunctival lesionto reduce the chance of recurrence.

In conclusion, ocular dirofilariasis is a rare condition with diverse and atypical presentations across various ocular and adnexal structures. Prompt and complete excision of the lesion, accompanied by meticulous histopathological evaluation, is essential for guiding appropriate management and ensuring favorable outcomes. Minimizing risk factors, such as avoiding exposure to wildlife and refraining from swimming in untreated freshwater, may help reduce the likelihood of infection.

## Data Availability

No datasets were generated or analysed during the current study.
